# Antimicrobial Effectiveness of Clove Oil in Decontamination of Ready-to-Eat Spinach (*Spinacia oleracea* L.)

**DOI:** 10.3390/foods14020249

**Published:** 2025-01-14

**Authors:** Abigail A. Armah, Kelvin F. Ofori, Kenisha Sutherland, Emmanuel Otchere, Winter A. Lewis, Wilbert Long

**Affiliations:** College of Agriculture, Science and Technology, Delaware State University, 1200 North DuPont Highway, Dover, DE 19901, USA; kfofori22@students.desu.edu (K.F.O.); ksutherland20@students.desu.edu (K.S.); eotchere22@students.desu.edu (E.O.); walewis19@students.desu.edu (W.A.L.)

**Keywords:** clove oil, essential oil, natural antimicrobial compounds, ready-to-eat produce, produce washing, *Escherichia coli*, *Staphylococcus aureus*, *Shigella flexneri*, *Salmonella* Typhimurium, *Salmonella enterica*

## Abstract

Due to an increased demand for natural food additives, clove oil was assessed as a natural alternative to chemical disinfectants in produce washing. This study assessed the antimicrobial activity of 5 and 10% (*v*/*v*) clove oil-amended wash liquid (CO) using a zone of inhibition (ZIB) test and determined the time required to completely inactivate pathogenic bacteria using bacterial death curve analysis. A washing experiment was used to evaluate CO’s ability to inhibit bacterial growth on inoculated RTE spinach and in the wash water. The findings showed that *Shigella flexneri*, *Salmonella* Typhimurium, and *Salmonella enterica* recovery were completely inhibited within 5 min. *Escherichia coli* and *Staphylococcus aureus* recovery were completely inhibited at 10 and 30 min, respectively. The ZIB test showed that 5% CO had the highest inhibitory effect on both Salmonella strains and *E. coli* with approximately 10 mm ZIB diameter. Additionally, 5% CO completely inactivated all bacterial strains on spinach samples and in the wash water except for *S. aureus*. A total of 80 mg/L peracetic acid (PAA) resulted in >2log CFU/mL recovery on experimental washed samples. These findings suggest that 5% CO was highly effective in inhibiting microbial growth on RTE spinach, potentially contributing to sustainable food safety and shelf-life extension strategies.

## 1. Introduction

Foodborne-related infections remain a significant public health challenge, resulting in approximately 600 million cases and about 420,000 deaths globally each year [1]. Ready-to-eat (RTE) fresh produce (fresh fruits and vegetables) consumption, on the other hand, has increased significantly globally due to convenience and increasing awareness of their health and nutritional benefits [2,3]. Between 1961 and 2021, the global average per capita consumption of vegetables per person per year increased from 63.29 kg to 147.04 kg [4]. Moreover, previous studies have reported reduced risk of mortality from cardiovascular diseases associated with the increased consumption of fresh produce [5,6,7]. However, the increase in fresh produce consumption has been paralleled by an increase in foodborne-related outbreaks [2,3,8]. From 1998 to 2013, the percentage of outbreaks associated with fresh produce doubled from 8% in 1998–2001 to 16% in 2010–2013 [9]. A wide spectrum of pathogens including *Staphylococcus aureus*, *Escherichia coli*, *Salmonella* spp., *Listeria monocytogenes*, and *Clostridium perfringens* associated with fresh produce have been documented [10,11,12]. The RTE fresh produce processing chain involves various unit operations such as selection, cleaning, washing, trimming, peeling, cutting, shredding and slicing [13], sanitizing, and packing, capable of providing opportunities for cross-contamination, whereby small portions of contaminated RTE produce may contaminate large portions of processed RTE produce [2,13,14,15,16]. This together with the lack of a thermal kill step to eliminate pathogenic and spoilage bacteria as well as other microbes that may be present during processing makes RTE fresh produce more susceptible to microbial contamination and a potential major vehicle for foodborne illness outbreaks [3,17]. Additionally, the presence of microorganisms contributes greatly to shelf-life reduction, resulting in substantial economic losses and deterioration of produce quality and acceptability [18,19,20].

Produce washing, a key step in RTE vegetable processing, is a major procedure in reducing the microbial load of fresh produce and largely determines the microbiological quality and shelf-life of RTE fresh-cut produce [2,21,22]. The RTE vegetable process water is recognized as an important potential source of cross-contamination with coliforms and human enteric pathogens, thereby contributing to the potential development of foodborne illnesses [2,23]. Chemical disinfectants such as chlorine or peracetic acid (PAA) are typically added to the RTE process water to maintain its microbiological quality [21,24,25]. In the fresh-cut produce industry, chlorine and chlorine-derived compounds, such as sodium hypochlorite (NaOCl), calcium hypochlorite (Ca(OCl)_2_), and chlorine gas (Cl_2_), remain the most commonly used chemical disinfectants as they are inexpensive and effective in reducing microbial load in the wash water [21,22,23]. The use of chlorine-based disinfectants has, however, come under scrutiny due to the potential to react with organic matter to form disinfection by-products (DBPs) and accumulate on washed produce, posing a potential health risk to consumers [21,23,26,27]. In some cases, peracetic acid is used as a sanitizing agent for fresh produce due to its effectiveness and less DBP compared to chlorine and chlorine-derived products [16,28,29].

Recently, clove essential oil (CO) has been used as a natural food preservative and colorant owing to its antibacterial and health-promoting properties [30]. Numerous studies have demonstrated strong antimicrobial properties of some plant-based essential oils [31,32,33,34]. A study by Barbosa et al. [35] assessing the antimicrobial activity of six essential oils, lemongrass, oregano, marjoram, thyme, ginger, basil, and clove, demonstrated that clove essential oil exhibited the highest antimicrobial activity. Much attention has been drawn to clove essential oil due to its high bioactive compound composition such as phenolics (eugenol) and terpenes (humulene and caryophyllene), among others with eugenol (4-allyl-2-methoxyphenol) being the most important and major composition of clove essential oil, responsible for its strong antimicrobial and pathogen inhibition properties [30,31,36,37,38]. Previous studies have demonstrated the ability of CO as well as its main bioactive component, eugenol, to inhibit microbial growth: *E. coli* [34,39,40]; *Klebsiella pneumoniae* [39,40]; *S. aureus* [34,40,41,42]; *Campylobacter jejuni* [42]; *Shigella* [42]; and *Salmonella typhi*, among others [42,43]. This occurs as a result of the disruption of bacterial cell membranes, leading to increased permeability, leakage of intracellular components such as ATP, and eventual cell death [42,43]. Additionally, clove essential oil prevents DNA synthesis and replication, inhibiting the expression of virulence factors and biofilm formation in bacterial cells which are crucial for their resistance and survival [44]. Other studies have reported the shrinkage and lysis of bacterial cell membranes and inhibition of β-lactamase production in bacteria such as *E. coli* [39,43]. Using eugenol, the main antimicrobial constituent of clove essential oil, ref. [30] reported the inhibition of biofilm formation in *E. coli* and *S. aureus* distortion, wrinkling, and serious alteration to *S. aureus* cells with the leakage of cytoplasmic content, increased oxidative stress to *S. aureus* cells as evidenced by high reactive oxygen species (ROS) production, as well as the inhibition of DNA synthesis after exposure to eugenol for 8 h [30]. Li et al. [45] reported morphological damage, significant leakage of intracellular content, and inhibited tricarboxylic acid cycle pathway, leading to inhibited respiratory metabolism when *S. aureus* was exposed to clove essential oil [33]. In a study evaluating clove essential oil’s influence based on concentration, temperature, and presence of organic matter, a 5-log decrease in *E. coli* population was reported by [33] when 0.4% of essential oil was applied at 21 °C. The inhibitory effects of eugenol extract obtained from cloves on the growth of resistant *Helicobacter pylori* strains were also demonstrated by [46]. Thus, clove essential oil has demonstrated strong antimicrobial properties and may serve as a natural and sustainable alternative to chemical antimicrobial agents commonly used in the RTE produce industry, reducing the accumulation of DBPs on RTE fresh produce and improving the microbiological quality of produce wash water. This study aimed to assess the effectiveness of CO-amended wash water in the decontamination of RTE fresh produce, particularly spinach, the time taken to completely inactivate specific bacterial species, and the microbiological quality of RTE wash water using the Nomad IoT smart microbiology device.

## 2. Materials and Methods

### 2.1. Antimicrobial Compounds

The clove essential oil (CO) used in this study was of commercial grade and purchased from Holistic Herbal Solutions (Grove City, OH, USA). The CO was 100% pure and extracted by steam distillation. The CO treatment was prepared by dilution with sterilized water to concentrations of 5 and 10 percent (*v*/*v*) and stirred for ~1 min to mix both liquids immediately before the start of each experiment.

### 2.2. Microorganisms

The bacterial strains (*Escherichia coli* k12 (cat# 155068), *Staphylococcus aureus* (cat# 155554A 705/3760), *Shigella flexneri* (cat# 155470A 705/3760), *Salmonella* Typhimurium (cat# 155351A 705/3760), and *Salmonella enterica* (cat# 155350A 705/3760)) were purchased from Carolina Biological Supply Co. (Burlington, NC, USA), activated, and stored at −80 °C as glycerol stock cultures. Subcultures were prepared by the aseptic inoculation of tryptic soy broth (TSB) (Carolina Biological Supply Co., Burlington, NC, USA) with a loop full of each bacterial strain and incubated for 24 h at 37 °C.

### 2.3. Zone of Inhibition

Tryptic soy agar (TSA) (Carolina Biological Supply Co. Burlington, NC, USA) was inoculated with 1 mL of each bacterial strain using the spread plate method. Subsequently, 5 mm filter paper disks were filled with 20 µL of each CO concentration (5% and 10%) and placed on appropriately labeled TSA plates inoculated with each bacterial strain. The inoculated TSA plates were incubated at 37 °C for 24 h, after which the clear zone of inhibition (ZIB) diameter around each filter paper disk was measured in mm. This was carried out in triplicate. Sterilized water (H_2_O) was used as a negative control, and peracetic acid (PAA) served as the positive control.

### 2.4. Bacterial Death Curve

The bactericidal effect of CO was assessed according to procedures previously described by [47]. Briefly, 1 mL of 5% CO was added to test tubes containing 9 mL of inoculum for each bacterial strain. Inoculum samples were obtained at time intervals of 0, 5, 10, 20, and 30 min, and the antimicrobial effect of CO was neutralized by serial dilution with peptone water. Neutralized dilutions were plated on selective media (xylose lysine deoxycholate (XLD) agar for *S. enterica*, *S.* Typhimurium, and *S. flexneri*; MacConkey agar for *E. coli*; and Mannitol Salt agar for *S. aureus*). The plates were incubated at 37 °C for 24 h, and data from enumeration were used to plot the bacterial death curve. The bacterial death curve experiment was performed in three replicates for each bacterial strain. Peracetic acid (80 mg/L) was used as a control.

### 2.5. RTE Washing Experiment

The method for the washing experiment involved modifications to a procedure previously described by [48]. The inoculum (50 µL) for each bacterial strain was pipetted and gently deposited in droplets using a micropipette onto the surface of the spinach samples. The bacteria were allowed to attach to the spinach surface by air-drying the inoculated spinach samples for 2 h. Subsequently, the inoculated spinach samples were washed with 200 mL of CO wash liquid for 5 min at 1500 RPW (washing machine spin speed) (portable washing machine GRINCHAT X-1, Frankfort, KY, USA), after which the washed spinach samples were macerated and 100 mL of the effluent obtained was plated on selective media at 37 °C for 24 h. The spinach wash water and the effluents from unwashed inoculated spinach samples were plated independently on selective media at 37 °C and incubated for 24 h. The plates were enumerated after incubation and the data obtained were analyzed. The experiment was performed in triplicate, and three spinach leaves were used per replicate. Peracetic acid (PAA) and sterilized water (H_2_O) were used as controls in separate washers.

### 2.6. RTE Total Bacteria Survival in CO Wash Water

Uninoculated spinach samples were washed with 5% CO-amended wash liquid for 5 min. Sterilized water and PAA served as the controls. Using the Nomad IoT Dip Sampler, the total bacterial counts in the spinach wash water were assessed for 144 h and yeast and mold growth for 96 h following the manufacturer’s instructions.

### 2.7. Sensory Evaluation

A sensory evaluation was conducted with an untrained sensory panel (*n* = 30) to evaluate the potential consumer acceptability of the washed spinach samples. At the start of the sensory evaluation, written informed consent was obtained from each participant. Using the five-point hedonic scale, quality attributes of aroma, texture, color, and overall acceptability of the uninoculated spinach samples washed with the CO-amended wash liquid were evaluated (Please refer to the Supplementary Materials for sensory evaluation sheet). Sterilized water and PAA served as controls for the sensory evaluation. Each study participant received one spinach leaf for each treatment group.

### 2.8. Statistical Analysis

The Statistical Package for Social Sciences (SPSS) version 29.0 (SPSS Inc., Chicago, IL, USA) and Microsoft Excel 2019 (Microsoft Inc., Redmond, WA, USA) were used for data analysis. The results were reported as mean values ± standard deviations. A one-way analysis of variance (ANOVA), followed by Tukey’s post hoc analysis, was used to compare logarithmic values and determine the statistical significance of the mean values. Results were considered statistically significant at *p* < 0.05.

## 3. Results

### 3.1. Zone of Inhibition

Table 1 shows the inhibitory effects of CO at various concentrations (5 and 10% *v*/*v*) on *E. coli*, *S. aureus*, *S. flexneri*, *S. enterica*, and *S.* Typhimurium (Appendix A). Except for the samples treated with H_2_O, noticeable ZIBs were observed for all treatment concentrations with both PAA and CO. For *E. coli*, *S. flexneri*, and *S. enterica*, 10% CO had the greatest ZIB compared to 80 mg/L PAA with ZIB values of 11.33 ± 0.58, 10.33 ± 0.58, and 11.33 ± 0.58 mm, respectively (*p* < 0.05). In contrast, PAA at both 60 and 80 mg/L had the greatest ZIB for *S. aureus* compared with CO (*p* < 0.05). No significant difference was observed in the inhibitory effects of CO and PAA on *S.* Typhimurium.

### 3.2. Bacterial Death Curve

**Figure 1:**
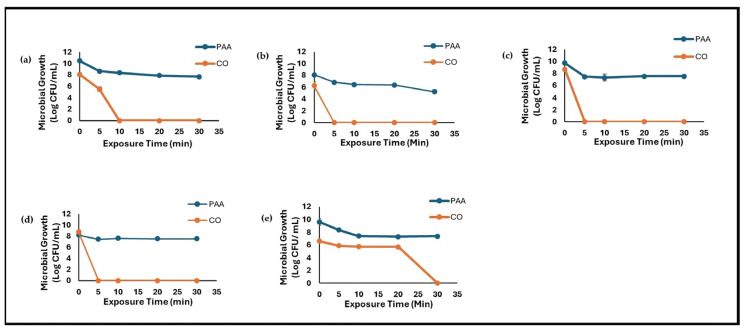
Reduction in bacterial recovery at 0, 5, 10, 20, and 30 min of exposure to CO and PAA. Values are recorded as means ± standard deviation (*n* = 3). (**a**) *Escherichia coli*; (**b**) *Shigella flexneri*; (**c**) *Salmonella enterica*; (**d**) *Salmonella* Typhimurium; (**e**) *Staphylococcus aureus*.

The effect of 5% CO on the survival of five bacterial species (*E. coli*, *S. aureus*, *S. flexneri*, *S. enterica*, and *S.* Typhimurium) compared to the effect of 80 mg/L PAA is shown in Figure 1a–e. The initial bacterial counts for *E. coli*, *S. enterica*, and *S.* Typhimurium were approximately 10log CFU/mL and ~8log CFU/mL for *S. flexneri* and *S.* Typhimurium for the PAA treatment group. For CO, an initial population of ~9log CFU/mL was recorded for *S. enterica*, *S.* Typhimurium, and *E. coli*, while *S. flexneri* and *S. aureus* recorded ~6.2 and 6.6log CFU/mL, respectively, at time point zero. At 5 min of exposure to CO, the recovery of *S. flexneri*, *S. enterica*, and *S.* Typhimurium declined to zero (0.00 ± 0.00log) with no further bacterial recovery recorded after the 5 min exposure time. *E. coli* recovery decreased by 2.6log at 5 min with no further recovery after 10 min of exposure to CO. On the contrary, bacterial recovery for the PAA treatment group at 5 min declined by 1.82, 1.24, 2.27, 0.73, and 1.26log CFU/mL for *E. coli*, *S. flexneri*, *S. enterica*, *S.* Typhimurium, and *S. aureus*, respectively. Unlike the results recorded for the CO treatment groups, bacterial recovery for the PAA treatment groups remained constant with no further significant decrease in recovery for *E. coli*, *S. enterica*, and *S.* Typhimurium after the 10 min exposure time, while *S. flexneri* declined by 1.73 and 2.8 logs, respectively, at 20 and 30 min, and *S. aureus* declined by 2.2 log at 10 min of exposure to PAA. Overall, CO had the greatest inhibitory effect as it completely inactivated *S. flexneri*, *S. enterica*, and *S.* Typhimurium at 5 min, *E. coli* at 10 min, and *S. aureus* at 30 min.

### 3.3. RTE Washing Experiment

**Figure 2:**
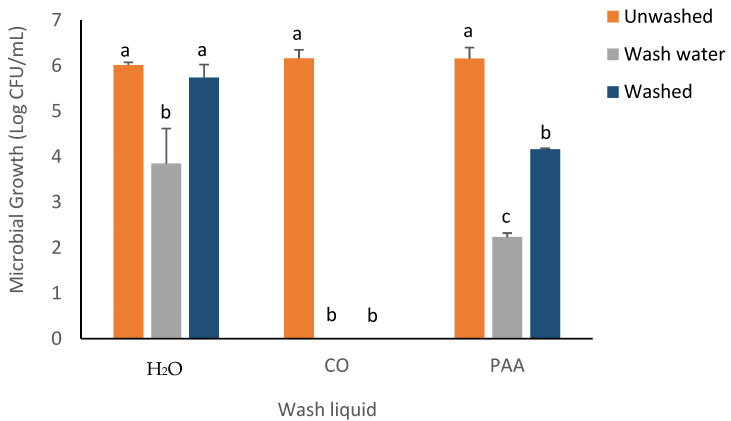
Recovery of *E. coli* K-12 from inoculated spinach, washed, unwashed, and wash water after 5 min washing with NEWL and CPWL. Values are recorded as mean ± standard deviation (*n* = 3). Different letters (a, b, c) above each bar indicate significant differences within treatment groups. Appropriate areas within the graph with no bars represent treatment samples with no microbial growth.

**Figure 3:**
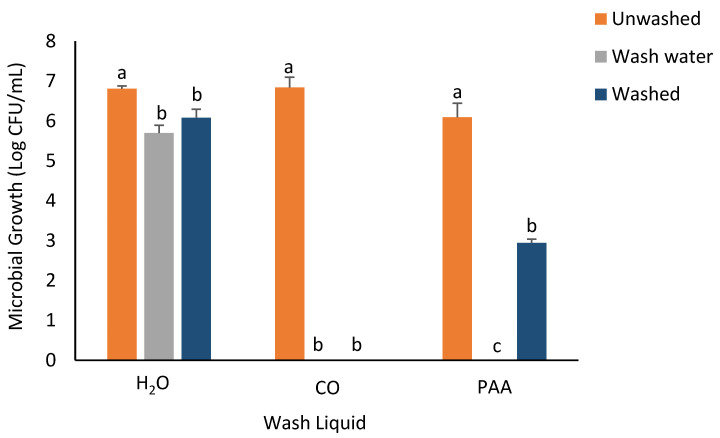
Recovery of *S. flexneri* from inoculated spinach, washed, unwashed, and wash water after 5 min washing with H_2_O, CO, and PAA. Values are recorded as mean ± standard deviation (*n* = 3). Different letters (a, b, c) above each bar indicate significant differences within treatment groups. Appropriate areas within the graph with no bars represent treatment samples with no microbial growth.

**Figure 4:**
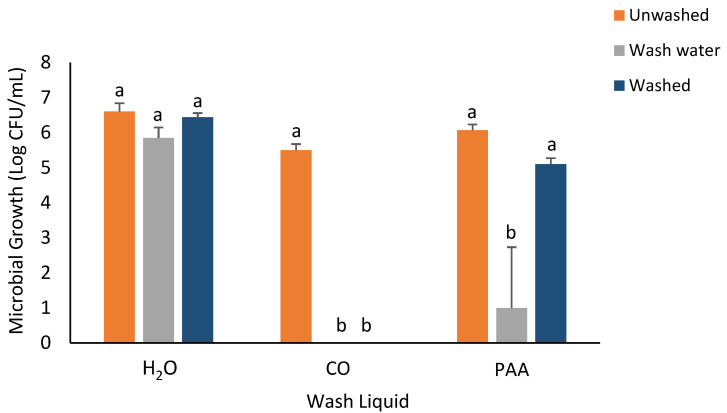
Recovery of *S. enterica* from inoculated spinach, washed, unwashed, and wash water after 5 min washing with H_2_O, CO, and PAA. Values are recorded as mean ± standard deviation (*n* = 3). Different letters (a, b) above each bar indicate significant differences within treatment groups. Appropriate areas within the graph with no bars represent treatment samples with no microbial growth.

**Figure 5:**
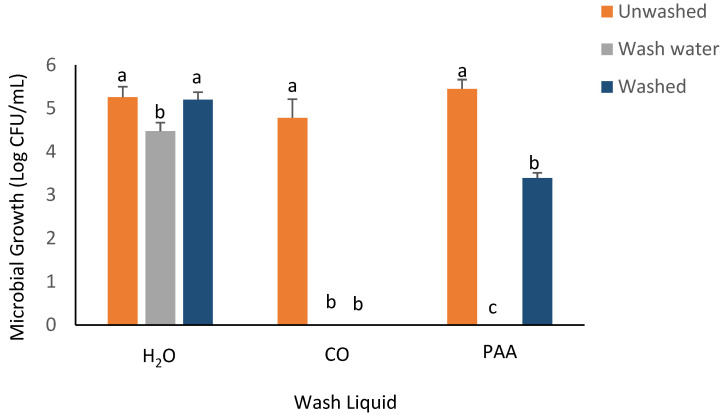
Recovery of *S.* Typhimurium from inoculated spinach, washed, unwashed, and wash water after 5 min washing with H_2_O, CO, and PAA. Values are recorded as mean ± standard deviation (*n* = 3). Different letters (a, b, c) above each bar indicate significant differences within treatment groups. Appropriate areas within the graph with no bars represent treatment samples with no microbial growth.

**Figure 6:**
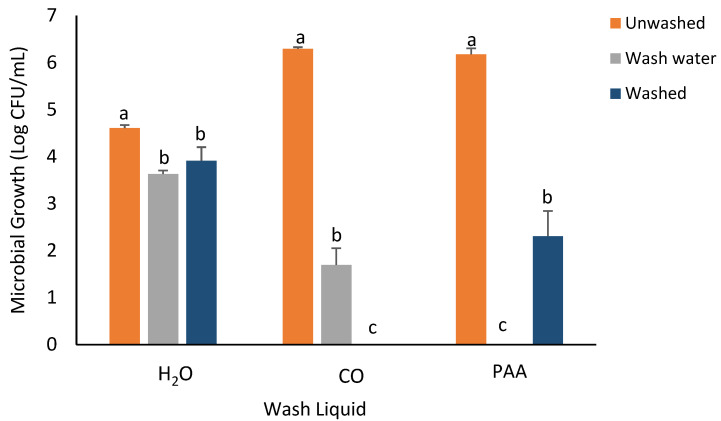
Recovery of *S. aureus* from inoculated spinach, washed, unwashed, and wash water after 5 min washing with H_2_O, CO, and PAA. Values are recorded as mean ± standard deviation (*n* = 3). Different letters (a, b, c) above each bar indicate significant differences within treatment groups. Appropriate areas within the graph with no bars represent treatment samples with no microbial growth.

The recovery of inoculated bacterial strains on spinach samples was evaluated using the RTE washing experiment, with the results outlined in Figure 2, Figure 3, Figure 4, Figure 5 and Figure 6. No significant difference was observed in *E. coli* recovery for the unwashed (6.01 ± 0.06log CFU/mL) and washed (5.74 ± 0.28log CFU/mL) samples when the H_2_O-only wash liquid was utilized. The *Escherichia coli* population decreased significantly in the washed inoculated spinach samples for the PAA and CO treatment groups. The CO wash liquid had the greatest effect in inhibiting *E. coli* recovery, as no bacterial growth was observed in the exudates of the washed spinach samples or the wash water when the CO wash liquid was utilized. For PAA, there was a 2.67log CFU/mL decrease in the *E. coli* population on the washed spinach samples and a 2.35log CFU/mL decrease in the wash water (Figure 2).

For *S. flexneri* (Figure 3), similar to observations for *E. coli*, the greatest reduction in the recovery of bacterial populations was recorded in the CO treatment group. No *S. flexneri* counts were observed on the washed spinach samples and the wash water when CO wash liquid was utilized. The least decrease in the recovery of *S. flexneri* in washed spinach samples was observed when H_2_O was used as the wash liquid (0.73log decrease). The PAA treatment group showed an average of 3.15log decrease in *S. flexneri* recovery, while no recovery was observed in the wash water.

Concerning *S. enterica*, there was no difference in bacterial recovery when H_2_O was used as the wash liquid. As indicated in Figure 4, the CO wash liquid had the greatest effect in inhibiting the recovery of *S. enterica* in the washed inoculated spinach samples, and no bacterial growth was observed in the wash water. No significant difference was recorded in the recovery of *S. enterica* in washed inoculated spinach samples for the PAA-treated samples.

Similarly to *S. enterica*, there was no difference in washed and unwashed spinach samples inoculated with *S.* Typhimurium when H_2_O was utilized (Figure 5). The greatest reduction in the recovery of *S.* Typhimurium was recorded for the CO treatment group as no visible bacterial growth was observed for the washed samples nor the wash water. A significant decrease (*p* < 0.05) in the recovery of *S.* Typhimurium was observed in both washed inoculated samples and wash water when PAA was utilized. For the washed spinach samples, the recovery of *S.* Typhimurium decreased by 2.06 log for the PAA treatment group. No bacterial recovery was observed in the PAA wash water.

The CO wash liquid had the greatest effect in inhibiting the recovery of *S. aureus* as no visible bacterial recovery was recorded for the washed spinach samples, and a 4.60 log decrease was observed in the wash water (*p* < 0.05). In the case of PAA, there was a significant decrease in *S. aureus* recovery for both washed and unwashed samples. *Staphylococcus aureus* recovery decreased by 3.87 log in the washed samples for PAA, while no bacterial recovery was reported in the PAA wash water. While there was a 0.70 log decrease in bacterial recovery for the washed spinach samples in the H_2_O treatment group, no difference was recorded in bacterial recovery for the wash water (Figure 6).

### 3.4. Nomad IoT

**Figure 7:**
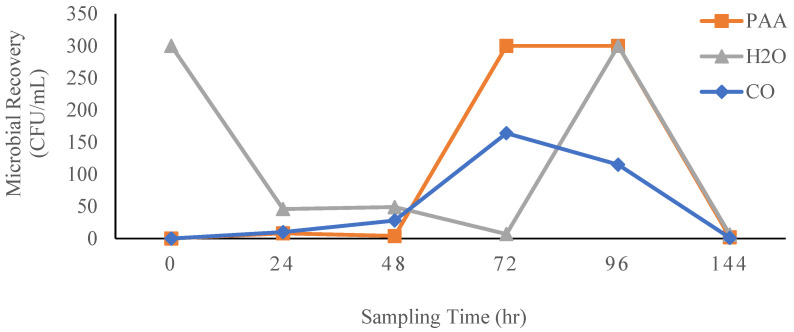
Recovery of uninoculated bacterial populations in spinach wash water after washing with H_2_O, PAA, and CO.

**Figure 8:**
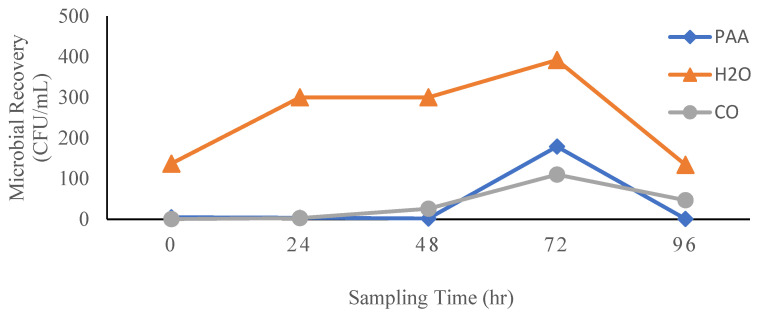
Recovery of uninoculated yeast and mold populations in spinach wash water after washing with H_2_O, PAA, and CO.

Figure 7 and Figure 8 illustrate the graphs for the recovery of total bacteria, yeast, and mold populations obtained using the Nomad IoT device in spinach wash water after washing with H_2_O, PAA, and CO. Compared to H_2_O, which served as a negative control, and PAA, which served as a positive control, a peak in the recovery of total bacteria for CO was recorded after 72 h (164 CFU/mL), and this was lower compared to the peak bacterial population recorded for PAA (after 72 h) and H_2_O immediately after washing (0 h) and after 96 h (>300 CFU/mL).

Yeast and mold growth remained consistently low for samples collected within the first 48 h when CO wash liquid was utilized (Figure 8). A peak in the recovery of yeast and mold was recorded after 72 h (110 CFU/ mL) for CO spinach wash water. This was, however, lower than yeast and mold growth recorded in wash water samples collected for PAA (179 CFU/mL) and for H_2_O (392 CFU/mL) after 72 h. The greatest yeast and mold recovery was observed in the H_2_O wash liquid samples as the microbial growth values remained consistently high throughout the 96 h.

To evaluate consumer acceptability for spinach samples washed with CO, PAA, and H_2_O, sensory evaluations were conducted using uninoculated spinach samples washed with PAA, H_2_O, and CO for quality attributes of color, texture, aroma, and overall acceptability (Table 2). There was no difference in consumer acceptability for each of the washed samples with regard to color (*p* > 0.05). The sensory evaluation participants preferred the texture and aroma of spinach samples washed with PAA and H_2_O over spinach samples washed with CO (*p* < 0.05). For overall acceptability, spinach samples washed with CO were less acceptable to the study participants than samples washed with PAA and H_2_O.

## 4. Discussion

Due to the high demand for natural antimicrobials, their health benefits, and their ability to impede the growth of bacteria, clove essential oil was evaluated for its effectiveness in the decontamination and shelf-life extension of RTE produce. Essential oils are known to contain a range of bioactive compounds including flavonoids and phenolics (such as eugenol in clove oil), which inhibit the survival and growth of harmful bacterial populations [31,34,39,49]. Moreover, the use of spices as preservatives and colorants in the food industry is becoming common; an example of which is clove oil, a great substitute for chemical preservatives due to its strong antimicrobial properties, aroma, and safety [36].

The results show that clove essential oil exhibits strong dose-dependent antimicrobial activity with inhibitory effects mostly greater than that of 80 mg/L peracetic acid. The zone of inhibition test showed ZIB values ranging from 7.67 to 11. 33 mm for CO and 8.33 to 19.00 mm for PAA. Based on the results of the zone of inhibition test, *E. coli* and *S. enterica* were most susceptible to the inhibitory effects of CO with zone of inhibition values of 11.33 mm for both *E. coli* and *S. enterica*, while *S. aureus* recorded the least inhibitory effect with ZIB values of 7.67 and 9.00 mm, respectively, for 5% and 10% CO. Similar ZIB values were reported by [50] when similar clove oil concentrations extracted from the stem were tested on *S. aureus*. In contrast to the zone of inhibition of CO, PAA had the strongest inhibitory effect on *S. aureus* with ZIB values of 16.67 and 19.00 mm for 60 and 80 mg/L PAA, respectively.

Similarly, with the bacterial death curve, except for *S. aureus* and *E. coli*, all bacterial strains tested were completely inactivated with no visible bacterial growth after 5 min of exposure to 5% CO. With regard to *E. coli*, recovery significantly decreased at 5 min and no visible growth was observed at 10 min. Although the recovery of *S. aureus* decreased by only 0.73 log at 5 min of exposure to 5% CO, the bacterial population was completely inactivated with no visible growth at 30 min. This was unsurprising as similar results were observed in the ZIB test where CO at 5% and 10% had the least inhibitory effect on *S. aureus.* Similar results were reported by [51] in their study to evaluate the antimicrobial activity of essential oils against human pathogens, where Gram-negative bacteria showed better sensitivity to clove essential oil’s antimicrobial activity than Gram-positive bacteria.

The response of the various bacterial strains to the antimicrobial compounds may be attributed to the difference in their cell wall structure. Gram-positive bacteria generally lack an outer membrane and have a thick peptidoglycan layer, which is porous and more permeable to hydrophilic compounds [52,53]. Gram-negative bacteria on the other hand have a more complex cell structure composed of a thin peptidoglycan layer and an outer membrane with thick lipopolysaccharides, which acts as an asymmetric lipid bilayer and a permeability barrier to antimicrobial compounds [52,54]. Thus, PAA, which is more hydrophilic, could easily penetrate the cell wall of the Gram-positive *S. aureus*, increasing its sensitivity, thereby resulting in slightly higher ZIBs compared to the Gram-negative bacterial strains (*E. coli*, *S. flexneri*, *S. enterica*, and *S.* Typhimurium).

On the other hand, even though Gram-negative bacteria are generally more difficult to inactivate due to the presence of a more resistant outer membrane containing lipopolysaccharide and their efflux pumps, among others [52], the eugenol in clove essential oil acts as a membrane permeabilizer, increasing the susceptibility of resistant Gram-negative bacterial strains [54]. Additionally, the hydrophobic nature of eugenol in clove essential oil allows it to pass through the lipopolysaccharide outer membrane, disrupting its integrity, leading to increased permeability of the membrane and leakage of cellular content, such as ATP and ions essential for cellular function [49,55,56]. This is in line with the findings reported by [49,55,56] which reported a disruption in bacterial cell membrane integrity, resulting in a pronounced increase in the leakage of bacterial proteins and DNA caused by eugenol [56]; the shrinkage of *Shigella flexneri* cells as a result of severe damage from increased cell permeability from exposure to eugenol [49]; and dose-dependent damage to *E. coli* cell membrane integrity and morphology, resulting in increased permeability caused by exposure to eugenol.

Similarly to findings from the literature [34,39,43], this study demonstrated that clove essential oil has the potential to inhibit the survival and growth of spoilage and pathogenic bacterial populations on RTE produce, particularly spinach. Washing the spinach samples independently with CO and PAA resulted in a significant decrease in the bacterial population on washed spinach samples and in wash water, while washing with H_2_O resulted mostly in no significant decrease in bacterial recovery in the wash water. Since the PAA and CO wash liquids had antimicrobial properties, they were able to inhibit the recovery of the bacterial strains in the wash water, resulting in reduced bacterial populations both on the washed spinach and in the wash water. While the CO wash liquid was very effective and resulted in no bacterial growth on both the spinach samples and in the wash water for all the Gram-negative bacteria (*E. coli*, *S. flexneri*, *S. enterica*, and S. Typhimurium), *S. aureus* recovery in the wash water was not completely inhibited. This may be due to the difference between the cell wall structure of Gram-negative and Gram-positive bacteria. Gram-positive bacteria generally lack an outer membrane and have a thick peptidoglycan layer, which is porous and more permeable to hydrophilic compounds [52,53]. On the other hand, Gram-negative bacteria have a more complex cell structure composed of a thin peptidoglycan layer and an outer layer with thick lipopolysaccharides, which acts as an asymmetric lipid bilayer and a permeability barrier to antimicrobial compounds [52,54]. Due to its hydrophobic nature, CO diffuses more easily through the hydrophobic lipid bilayer outer membrane of the Gram-negative bacteria, disrupting its integrity and permeability, compared to Gram-positive bacteria, which selectively allows small hydrophobic molecules to pass through the thick peptidoglycan layer but is more resistant to hydrophobic compounds [54]. Additionally, the eugenol in clove essential oil acts as a membrane permeabilizer, increasing the susceptibility of resistant Gram-negative bacterial strains [54].

Washing with water only provides minimal physical removal of bacteria from surfaces [57]. With regard to the H_2_O treatment group, the washing process dislodged and removed some of the *S. flexneri* and *S. aureus* attached to the surface of the spinach samples. However, since H_2_O had no antimicrobial effect, it was unable to inactivate the bacteria in the wash water, resulting in no significant decrease in wash water bacterial populations for *S. aureus* and *S. flexneri*. On the other hand, bacteria such as *E. coli* and *Salmonella* can attach themselves strongly to the surface and crevices of leafy vegetables and form biofilms, making them difficult to dislodge with just water [58,59], which may lead to no significant decrease in *E. coli*, *S. enterica*, and *S.* Typhimurium in the washed spinach samples being observed.

The Nomad smart microbiology IoT test device is a smart device which allows for pen-free on-site sampling of liquids and surfaces [60]. It detects and enumerates aerobic bacteria, yeasts, and molds in liquids and on surfaces, eliminating the need for long laboratory procedures in detecting and monitoring microbial contamination during processing [61]. When microbial recovery in spinach wash water was monitored for 144 h (6 days) using the Nomad smart microbiology IoT test device, recovery of total bacteria was observed to be highest in the H_2_O treatment group in wash water samples collected right after the washing process. This may be attributed to the lack of sanitizing agents in the H_2_O treatment group, which resulted in initial bacterial populations on the spinach surfaces not being effectively inactivated. The microbial populations in the wash water could thus multiply with time and potentially spread further across different spinach surfaces during subsequent washes. Compared to H_2_O and PAA, the microbial population in the spinach wash water remained low when CO wash liquid was used. This can be attributed to the antimicrobial properties of the clove essential oil as demonstrated in previous studies [39,51].

Sensory evaluation of the samples revealed that the study participants preferred spinach samples washed with H_2_O more compared to samples washed with CO due to the strong odor and soft texture of the CO-treated samples, although CO was more effective in inactivating microbial populations on spinach samples and in the wash water. The strong odor could be attributed to the high concentration of eugenol, the main antimicrobial compound in clove oil, which has a strong spicy and pungent smell [62]. Additionally, the same properties responsible for the antimicrobial and antioxidant activity of eugenol in clove oil could potentially degrade the cell walls and structural components of the delicate spinach leaves when in contact with the CO wash liquid for an extended time. Post-treatment rinsing of the washed spinach after the initial wash with CO wash liquid may reduce the strong odor of eugenol and reduce its effect on the texture of washed spinach samples. Additionally, the encapsulation of clove oil [63], reducing the clove oil concentration, as well as its combination with other essential oils with complementary antimicrobial properties [64] but with a milder odor could improve the consumer acceptability of CO-treated samples. With the current increase in consumer demand for natural preservatives in food processing due to health concerns associated with synthetic preservatives [65], coupled with clove oil’s strong antimicrobial properties, this will provide a safer natural alternative to chemical preservatives commonly used in produce washing and extend the shelf-life of fresh produce.

## 5. Conclusions

Overall, 5% CO significantly inhibited the recovery of both Gram-negative bacteria (*E. coli*, *S. enterica*, S. Typhimurium, and *S. flexneri*) and Gram-positive bacteria (*S. aureus*). The Gram-negative bacteria were more susceptible to the inhibitory effect of clove essential oil as *S. enterica*, *S.* Typhimurium, and *S. flexneri* were completely inactivated within 5 min and *E. coli* was completely inactivated within 10 min of exposure to clove essential oil. *Staphylococcus aureus* was completely inactivated by clove essential oil at 30 min. Additionally, washing inoculated spinach samples independently with CO and PAA wash liquids for 5 min significantly decreased the population of inoculated bacteria on the spinach samples; however, this was not the case for samples washed with H_2_O only. Compared to PAA, the CO wash liquid appeared to be more promising in inhibiting the growth of Gram-negative bacteria, although it resulted in less desirable sensory characteristics in the washed spinach samples. These results suggest the potential effectiveness of CO wash liquid in reducing fresh produce microbial contamination and cross contamination from produce wash water. Furthermore, it may reduce the accumulation of DBPs in produce wash water and serve as a natural alternative to synthetic preservatives in produce washing to enhance food safety. Based on the findings of this study, recommendations for future studies using lower concentrations of encapsulated clove essential oil to assess its long-term effectiveness as well as studies to assess the effectiveness of different mild natural preservatives with complementary antimicrobial properties in combination with clove oil are warranted. These will improve the sensory characteristics of fresh produce washed with clove oil wash liquid and provide a safe natural alternative to synthetic preservatives commonly used in the fresh produce industry.

## Figures and Tables

**Table 1:** Zones of inhibition for different bacterial strains and total bacteria from baby spinach after treatment with CO, PAA, and H_2_O.

	ZIB for Microbial Strain				
Treatment/Conc.	*Escherichia coli*	*Staphylococcus aureus*	*Shigella flexneri*	*Salmonella enterica*	*Salmonella* Typhimurium
H_2_O	0.00 ± 0.00 ^a^	0.00 ± 0.00 ^a^	0.00 ± 0.00 ^a^	0.00 ± 0.00 ^a^	0.00 ± 0.00 ^a^
PAA (mg/L)					
60	8.00 ± 0.00 ^b^	16.67 ± 4.16 ^c^	8.33 ± 0.58 ^b^	8.00 ± 0.00 ^b^	8.33 ± 1.53 ^b^
80	8.33 ± 0.58 ^b^	19.00 ± 3.60 ^c^	9.00 ± 1.00 ^b^	8.33 ± 0.58 ^b^	9.33 ± 1.53 ^b^
CO (% *v*/*v*)					
5	9.67 ± 1.15 ^bc^	7.67 ± 0.58 ^b^	8.33 ± 0.58 ^b^	10.00 ± 1.15 ^bc^	9.67 ± 4.62 ^b^
10	11.33 ± 0.58 ^c^	9.00 ± 1.00 ^b^	10.33 ± 0.58 ^bc^	11.33 ± 0.58 ^c^	9.33 ± 1.53 ^b^

Values are presented as mean ± standard deviation (*n* = 3). Different letters (a, b, c) superscripted to mean ± standard deviation indicate significant differences within each column. Statistical significance was defined as *p* ≤ 0.05.

**Table 2:** Consumer acceptability of quality attributes of spinach washed with PAA, H_2_O, and CO.

Quality Attribute	PAA	H_2_O	CO
Color	3.77 ± 1.15 ^a^	3.77 ± 1.12 ^a^	3.48 ± 1.18 ^a^
Texture	3.94 ± 0.96 ^a^	3.61 ± 1.20 ^a^	2.39 ± 1.31 ^b^
Aroma	3.45 ± 1.15 ^a^	3.77 ± 1.12 ^a^	2.45 ± 1.57 ^b^
Overall Acceptability	3.74 ± 0.93 ^a^	3.84 ± 1.16 ^a^	2.42 ± 1.38 ^b^

Values are recorded as means ± standard deviation. *n* ≥ 30. Different letters (a, b) superscripted to mean ± standard deviation indicate significant differences between treatment groups. Statistical significance was defined as *p* ≤ 0.05.

**Figure A1:**
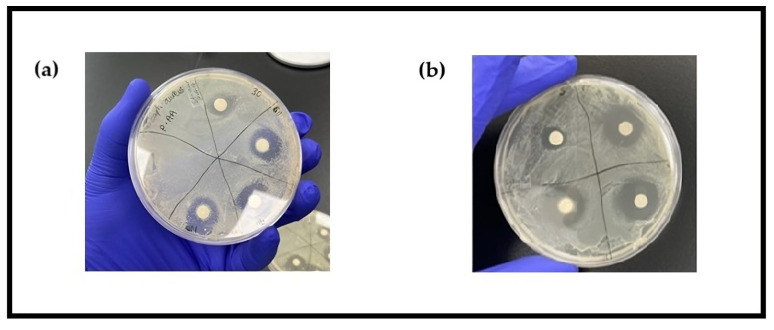
Sample images of zone of inhibition test. (**a**) PAA; (**b**) CO.

## Data Availability

No new data were created or analyzed in this study. Data sharing is not applicable to this article.

## Supplementary Materials

The following supporting information can be downloaded at: https://www.mdpi.com/article/10.3390/foods14020249/s1, Supplementary File: Sensory Evaluation Sheet: Evaluation of plant extracts for application in decontamination and shelf-life extension of ready-to-eat (RTE) produce.
