# Supplementary material for: Antimicrobial Effectiveness of Clove Oil in Decontamination of Ready-to-Eat Spinach (Spinacia oleracea L.)
Source: Foods. 2025 Jan 14;14(2):249. doi: 10.3390/foods14020249 (PMC11765317; doi:10.3390/foods14020249)
Supplement: Supplementary file 1 [file foods-14-00249-s001.zip › foods-3390436-supplementary.pdf]

Sensory Evaluation Sheet

Evaluation of plant extracts for application in decontamination and shelf-life extension of ready-to-eat (RTE) produce

Date _______________

Instructions:
You have been provided with five spinach samples. Please write the sample code for each sample in the space provided and assess each sample based on color, aroma, texture, and overall acceptability. After assessing each sample, please place an "x" mark on the point on the scale which best describes your feelings.

Sample code: ____________

**Score**	**Color**	**Texture**	**Aroma**	**General Acceptability**
1 – Dislike extremely				
2 – Dislike slightly				
3 – Neither like nor dislike				
4 – Like slightly				
5 – Like extremely				

Please provide brief reasons for your choice:
Color:

Texture:

Aroma:

General Acceptability:

Sample code: ____________

**Score**	**Color**	**Texture**	**Aroma**	**General Acceptability**
1 – Dislike extremely				
2 – Dislike slightly				
3 – Neither like nor dislike				
4 – Like slightly				
5 – Like extremely				

Please provide brief reasons for your choice:
Color:

Texture:

Aroma:

General Acceptability:

Sample code: ____________

**Score**	**Color**	**Texture**	**Aroma**	**General Acceptability**
1 – Dislike extremely				
2 – Dislike slightly				
3 – Neither like nor dislike				
4 – Like slightly				
5 – Like extremely				

Please provide brief reasons for your choice:
Color:

Texture:

Aroma:

General Acceptability:

Sample code: ____________

**Score**	**Color**	**Texture**	**Aroma**	**General Acceptability**
1 – Dislike extremely				
2 – Dislike slightly				
3 – Neither like nor dislike				
4 – Like slightly				
5 – Like extremely				

Please provide brief reasons for your choice:
Color:

Texture:

Aroma:

General Acceptability:

Sample code: ____________

**Score**	**Color**	**Texture**	**Aroma**	**General Acceptability**
1 – Dislike extremely				
2 – Dislike slightly				
3 – Neither like nor dislike				
4 – Like slightly				
5 – Like extremely				

Please provide brief reasons for your choice:
Color:

Texture:

Aroma:

General Acceptability:
